# Machine learning prediction in cardiovascular diseases: a meta-analysis

**DOI:** 10.1038/s41598-020-72685-1

**Published:** 2020-09-29

**Authors:** Chayakrit Krittanawong, Hafeez Ul Hassan Virk, Sripal Bangalore, Zhen Wang, Kipp W. Johnson, Rachel Pinotti, HongJu Zhang, Scott Kaplin, Bharat Narasimhan, Takeshi Kitai, Usman Baber, Jonathan L. Halperin, W. H. Wilson Tang

**Affiliations:** 1 Section of Cardiology, Baylor College of Medicine, Houston, TX USA; 2 Harrington Heart & Vascular Institute, Case Western Reserve University, University Hospitals Cleveland Medical Center, Cleveland, OH USA; 3 Department of Cardiovascular Diseases, New York University School of Medicine, New York, NY USA; 4 Robert D. and Patricia E. Kern Center for the Science of Health Care Delivery, Rochester, MN USA; 5 Division of Health Care Policy and Research, Department of Health Sciences Research, Mayo Clinic, Rochester, MN USA; 6 Department of Genetics and Genomic Sciences, Institute for Next Generation Healthcare, Icahn School of Medicine at Mount Sinai, New York, NY USA; 7 Levy Library, Icahn School of Medicine at Mount Sinai, New York, NY USA; 8 Division of Cardiovascular Diseases, Mayo Clinic, Rochester, MN USA; 9 Department of Cardiovascular Diseases, Icahn School of Medicine at Mount Sinai, Mount Sinai Hospital, Mount Sinai Heart, New York, NY USA; 10 Department of Cardiovascular Medicine, Heart and Vascular Institute, Cleveland Clinic, Cleveland, OH USA

**Keywords:** Computational biology and bioinformatics, Machine learning, Cardiovascular diseases

## Abstract

Several machine learning (ML) algorithms have been increasingly utilized for cardiovascular disease prediction. We aim to assess and summarize the overall predictive ability of ML algorithms in cardiovascular diseases. A comprehensive search strategy was designed and executed within the MEDLINE, Embase, and Scopus databases from database inception through March 15, 2019. The primary outcome was a composite of the predictive ability of ML algorithms of coronary artery disease, heart failure, stroke, and cardiac arrhythmias. Of 344 total studies identified, 103 cohorts, with a total of 3,377,318 individuals, met our inclusion criteria. For the prediction of coronary artery disease, boosting algorithms had a pooled area under the curve (AUC) of 0.88 (95% CI 0.84–0.91), and custom-built algorithms had a pooled AUC of 0.93 (95% CI 0.85–0.97). For the prediction of stroke, support vector machine (SVM) algorithms had a pooled AUC of 0.92 (95% CI 0.81–0.97), boosting algorithms had a pooled AUC of 0.91 (95% CI 0.81–0.96), and convolutional neural network (CNN) algorithms had a pooled AUC of 0.90 (95% CI 0.83–0.95). Although inadequate studies for each algorithm for meta-analytic methodology for both heart failure and cardiac arrhythmias because the confidence intervals overlap between different methods, showing no difference, SVM may outperform other algorithms in these areas. The predictive ability of ML algorithms in cardiovascular diseases is promising, particularly SVM and boosting algorithms. However, there is heterogeneity among ML algorithms in terms of multiple parameters. This information may assist clinicians in how to interpret data and implement optimal algorithms for their dataset.

## Introduction

Machine learning (ML) is a branch of artificial intelligence (AI) that is increasingly utilized within the field of cardiovascular medicine. It is essentially how computers make sense of data and decide or classify a task with or without human supervision. The conceptual framework of ML is based on models that receive input data (e.g., images or text) and through a combination of mathematical optimization and statistical analysis predict outcomes (e.g., favorable, unfavorable, or neutral). Several ML algorithms have been applied to daily activities. As an example, a common ML algorithm designated as SVM can recognize non-linear patterns for use in facial recognition, handwriting interpretation, or detection of fraudulent credit card transactions^1,2^. So-called boosting algorithms used for prediction and classification have been applied to the identification and processing of spam email. Another algorithm, denoted random forest (RF), can facilitate decisions by averaging several nodes. While convolutional neural network (CNN) processing, combines several layers and apples to image classification and segmentation^3–5^. We have previously described technical details of each of these algorithms^6–8^, but no consensus has emerged to guide the selection of specific algorithms for clinical application within the field of cardiovascular medicine. Although selecting optimal algorithms for research questions and reproducing algorithms in different clinical datasets is feasible, the clinical interpretation and judgement for implementing algorithms are very challenging. A deep understanding of statistical and clinical knowledge in ML practitioners is also a challenge. Most ML studies reported a discrimination measure such as the area under an ROC curve (AUC), instead of p values. Most importantly, an acceptable cutoff for AUC to be used in clinical practice, interpretation of the cutoff, and the appropriate/best algorithms to be applied in cardiovascular datasets remain to be evaluated. We previously proposed the methodology to conduct ML research in medicine^6^. Systematic review and meta-analysis, the foundation of modern evidence-based medicine, have to be performed in order to evaluate the existing ML algorithm in cardiovascular disease prediction. Here, we performed the first systematic review and meta-analysis of ML research over a million patients in cardiovascular diseases.

## Methods

This study is reported in accordance with the Preferred Reporting Information for Systematic Reviews and Meta-Analysis (PRISMA) recommendations. Ethical approval was not required for this study.

### Search strategy

A comprehensive search strategy was designed and executed within the MEDLINE, Embase, and Scopus databases from database inception through March 15, 2019. One investigator (R.P.) designed and conducted the search strategy using input from the study’s principal investigator (C.K.). Controlled vocabulary, supplemented with keywords, was used to search for studies of ML algorithms and coronary heart disease, stroke, heart failure, and cardiac arrhythmias. The detailed strategy is available from the reprint author. The full search strategies can be found in the supplementary documentation.

### Study selection

Search results were exported from all databases and imported into Covidence^9^, an online systematic review tool, by one investigator (R.P.). Duplicates were identified and removed using Covidence's automated de-duplication functionality. The de-duplicated set of results was screened independently by two reviewers (C.K. and H.V.) in two successive rounds to identify studies that met the pre-specified eligibility criteria. In the initial screening, two investigators (C.K. and H.V.) independently examined the titles and abstracts of the records retrieved from the search via the Covidence portal and used a standard extraction form. Conflicts were resolved through consensus and reviewed by other investigators. We included abstracts with sufficient evaluation data, including methodology, the definition of outcomes, and an appropriate evaluation matrix. Studies without any kind of validation (external validation or internal validation) were excluded. We excluded reviews, editorials, non-human studies, letters without sufficient data.

### Data extraction

We extracted the following information, if possible, from each study: authors, year of publication, study name, test types, testing indications, analytic models, number of patients, endpoints (CAD, AMI, stroke, heart failure, and cardiac arrhythmias), and performance measures ((AUC, sensitivity, specificity, positive cases (the number of patients who used the AI and were positively diagnosed with the disease), negative cases (the number of patients who used the AI and were negative with the AI test), true positives, false positives, true negatives, and false negatives)). CAD was defined as coronary artery stenosis > 70% using angiography or FFR-based significance. Cardiac arrhythmias included studies involving bradyarrhythmias, tachyarrhythmias, atrial, and ventricular arrhythmias. Data extraction was conducted independently by at least two investigators for each paper. Extracted data were compared and reconciled through consensus. In case studies which did not report positive and negative cases, we manually calculated by standard formulae using statistics available in the manuscripts or provided by the authors. We contacted the authors if the data of interest were not reported in the manuscripts or abstracts. The order of contact originated with the corresponding author, followed by the first author, and then the last author. If we were unable to contact the authors as specified above, the associated studies were excluded from the meta-analysis (but still included it in the systematic review). We also excluded manuscripts or abstracts without sufficient evaluation data after contacting the authors.

### Quality assessment

We created the proposed guidance quality assessment of clinical ML research based on our previous recommendation (Table 1)^6^. Two investigators (C.K. and H.V.) independently assessed the quality of each ML study by using our proposed guideline to report ML in medical literature (Supplementary Table S1). We resolved disagreements through discussion amongst the primary investigators or by involving additional investigators to adjudicate and establish a consensus. We scored study quality as low (0–2), moderate (2.5–5), and high quality (5.5–8) as clinical ML research.

**Table 1:** Proposed quality assessment of ML research for clinical practice.

**Algorithms**
Clarity of algorithms Propose new algorithms Select the proper algorithms Compare alternative algorithms
**Resources**
Reliable database/center Number of database/centers Number of samples (patients/images) Type and diversity of data
**Sufficient reported data**
Manuscript with sufficient supplementary information Letter or editor, short article, abstract Report baseline characteristics of patients
**Ground truth**
Comparison to expert clinicians Comparison to validated clinical risk models
**Outcome**
Assessment of outcome based on standard medical taxonomy External validation cohort
**Interpretation**
Report both discrimination and calibration metrics Report one or more of the following: sensitivity, specificity, positive, negative cases, balanced accuracy

### Statistical analysis

We used symmetrical, hierarchical, summary receiver operating characteristic (HSROC) models to jointly estimate sensitivity, specificity, and AUC^10^. ${Sen}_{i}$ and ${Spc}_{i}$ denote the sensitivity and specificity of the *i*th study. ${\sigma }_{Sen}^{2}$ is the variance of ${\mu }_{Sen}$ and ${\sigma }_{Spc}^{2}$ is the variance of ${\mu }_{spc}$.

$${\mu }_{Seni}=logit({Sen}_{i})$$

$${\mu }_{spci}=logit({Spc}_{i})$$

$$\left(\begin{array}{c}{\mu }_{Seni}\\ {\mu }_{spci}\end{array}\right)\sim N\left\{\left(\begin{array}{c}{\mu }_{Sen}\\ {\mu }_{Spc}\end{array}\right), \left(\begin{array}{cc}{\sigma }_{Sen}^{2}& {\sigma }_{SenSpc}\\ {\sigma }_{SenSpc}& {\sigma }_{Spc}^{2}\end{array}\right)\right.$$

The HSROC model for study* i* fits the following

$$logit({\pi }_{ij})=\left({\theta }_{i}+{\alpha }_{i}{X}_{ij}\right)\mathrm{exp}(-\beta {X}_{ij})$$

${\pi }_{i1}$ = ${Sen}_{i}$ and ${\pi }_{i0}$ =1- ${Spc}_{i}$. ${X}_{ij}=-\frac{1}{2}$ when no disease and ${X}_{ij}=\frac{1}{2}$ for those with disease. And ${\theta }_{i}$ and ${\alpha }_{i}$ follow normal distribution.

We conducted subgroup analyses stratified by ML algorithms. We assessed the performances of a subgroup-specific and statistical test of interaction among subgroups. We performed all statistical analyses using OpenMetaAnalyst for 64-bit (Brown University), R version 3.2.3 (Metafor and Phia packages), and Stata version 16.1 (Stata Corp, College Station, Texas). The meta-analysis has been reported in accordance with the Meta-analysis of Observational Studies in Epidemiology guidelines (MOOSE)^11^.

## Results

### Study search

**Figure 1:**
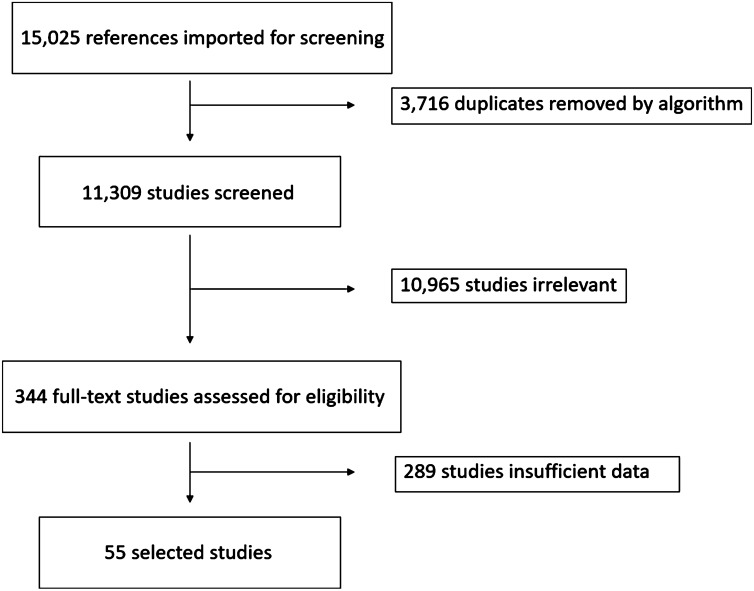
Study design. This flow chart illustrates the selection process for published reports.

The database searches between 1966 and March 15, 2019, yielded 15,025 results. 3,716 duplicates were removed by algorithms. After the screening process, we selected 344 articles for full-text review. After full text and supplementary review, we excluded 289 studies due to insufficient data to perform meta-analytic approaches despite contacting corresponding authors. Overall, 103 cohorts (55 studies) met our inclusion criteria. The disposition of studies excluded after the full-text review is shown in Fig. 1.

### Study characteristics

Table 2 shows the basic characteristics of the included studies. In total, our meta-analysis of ML and cardiovascular diseases included 103 cohorts (55 studies) with a total of 3,377,318 individuals. In total, 12 cohorts  assessed cardiac arrhythmias (3,144,799 individuals), 45 cohorts are CAD-related (117,200 individuals), 34 cohorts are stroke-related (5,577 individuals), and 12 cohorts are HF-related (109,742 individuals). The characteristics of the included studies are listed in Table 2. We performed post hoc sensitivity analysis, excluding each study, and found no difference among the results.

**Table 2:** Characteristics of the included studies.

First author	Analytic model	Sample	Indication	Imaging	Comparison	Database
**Cardiac arrhythmias**						
Alickovic et al. (2016)	RF	47	Arrhythmia detection	ECG	Five ECG signal patterns from MIT-BIH (normal (N), Premature Ventricular Complex (PVC), Atrial Premature Contraction (APC), Right Bundle Branch Block (RBBB) and Left Bundle Branch Block (RBBB)) and four ECG patterns from St. -Petersburg Institute of Cardiological Technics 12lead Arrhythmia Database (N, APC, PVC and RBBB)	St. Petersburg and MIT-BIH database
Au-Yeung et al. (2018)	5sRF, 10sRF, SVM	788	Ventricular arrhythmia	ICD Data		SCD-HeFT study
Hill et al. (2018)	Logistic-linear regression, SVM, RF	2,994,837	Development of AF/flutter in gen pop	Clinical data	ML compared with conventional linear statistical methods	UK Clinical Practice Research Datalink (CPRD) between 01–01-2006 and 31–12-2016 was undertaken
Kotu et al. (2015)	k-NN, SVM, RF	54	arrhythmic risk stratification of post MI patients	Cardiac MRI	Low LVEF and Scar versus textural features of scar	Single center
Ming-Zher Poh et al. (2018)	CNN	149,048	AF	ECG		Several publicly accessible PPG repositories, including the MIMIC-III critical care database,11 the Vortal data set from healthy volunteers12 and the IEEE-TBME PPG Respiratory Rate Benchmark data set.1
Xiaoyan Xu et al. (2018)	CNN	25	AF	ECG	MIT-BIH Atrial Fibrillation database	MIT-BIH Atrial Fibrillation Database
**Coronary artery disease**						
Araki et al. (2016)	SVM classifier with five different kernels sets	15	Plaque rupture prediction	IVUS	40 MHz catheter utilizing iMap (Boston Scientific, Marlborough, MA, USA) with 2,865 frames per patient (42,975 frames) and (b) linear probe B-mode carotid ultrasound (Toshiba scCNNer, Japan)	Single center
Araki et al. (2016)	SVM combined with PCA	19	Coronary risk assessment	IVUS		Single center
Arsanjani et al. (2013)	boosting algorithm	1,181	Perfusion SPECT in CAD	Perfusion SPECT	2 experts, combined supine/prone TPD	Single center
BaumCNN et al. (2017)	Custom-built algorithm	258	ctFFR in detecting relevant lesions		Invasive FFR determination of relevant lesions	the MACHINE Registry
Coenen (2018)	Custom-built algorithm	351	Invasive FFR / Computational flow dynamics based (CFD-FFR)	CT angiography	Invasive FFR / Computational flow dynamics based (CFD-FFR)	5 centers in Europe, Asia, and the United States
Dey et al. (2015)	boosting algorithm	37	Coronary CTA in ischemic heart disease patients to predict impaired myocardial flow reserve	CCTA	Clinical stenosis grading	Single center
Eisenberg et al. (2018)	boosting algorithm	1925	MPI in CAD	SPECT	Human visual analysis	The ReFiNE registry
Freiman et al. (2017)	Custom-built algorithm	115	CCTA in coronary artery stenosis	CCTA	Cardiac image analysis	The MICCAI 2012 challenge
Guner et al. (2010)	CNN	243	Stable CAD	Myocardial perfusion SPECT (MPS)	SPECT evaluation and human–computer interaction One expert reader who has 10 years of experience and six nuclear medicine residents who have two to four years of experience in nuclear cardiology took part in the study	Single center
Hae et al. (2018)	Logistic-linear regression, SVM, RF, boosting algorithm	1,132	Prediction FFR in stable and unstable angina patients	FFR, CCTA		Single center
Han et al. (2017)	Logistic-linear regression	252	Physiologically significant CAD	CCTA and invasive fractional flow reserve (FFR		The DeFACTO study
Hu (Xiuhua) et al. (2018)	Custom-built algorithm	105	Intermediate coronary artery lesions	CCTA	CCTA-FFR vs Invasive angiography FFR	Single center
Hu et al. (2018)	Boosting algorithm	1861	MPI in CAD	SPECT	True early reperfusion	Multicenter REFINE SPECT registry
Wei et al. (2014)	Custom-built algorithm	83	Noncalcified plaques (NCPs) detection on CCTA	CCTA		Single center
Kranthi et al. (2017)	Boosting algorithm	85,945	CCTA in CAD	CCTA	66 available parameters (34 clinical parameters, 32 laboratory parameters)	Single center
Madan et al. (2013)	SVM	407	Urinary proteome in CAD	global proteomic profile analysis of urinary proteome		Indian Atherosclerosis Research Study
Zellweger et al. (2018)	Custom-built algorithm	987	CAD evaluation	N/A	Framingham scores	The Ludwigshafen Risk and Cardiovascular Health Study (LURIC)
Moshrik Abd alamir et al. (2018)	Custom-built algorithm	923	ED patients with chest pain -CTA analysis	CT Angiography		Single center
Nakajima et al. (2017)	CNN	1,001	Previous myocardial infarction and coronary revascularization	SPECT	Expert consensus interpretations	Japanese multicenter study
Song et al. (2014)	SVM	208	Risk prediction in ACS	N/A		Single center
VanHouten et al. (2014)	Logistic-linear regression, RF	20,078	Risk prediction in ACS	N/A		Single center
Xiao et al. (2018)	CNN	15	Ischemic ST change in ambulatory ECG	ECG		Long-Term ST Database (LTST database) from PhysioNet
Yoneyama et al. (2017)	CNN	59	Detecting culprit coronary arteries	CCTA and myocardial perfusion SPECT		Single center
**Stroke**						
Abouzari et al. (2009)	CNN	300	SDH post-surgery outcome prediction	CT head		Single center
Alexander Roederer et al.(2014)	Logistic-linear regression	81	SAH-Vasospasm prediction	Passively obtained clinical data		Single center
Arslan et al. (2016)	Logistic-linear regression, SVM, boosting algorithm	80	Ischemic stroke	EMR		Single center
Atanassova et al. (2008)	CNN	54	Major stroke	Diastolic BP	2 CNNs compared	Single center
Barriera et al. (2018)	CNN	284	Stroke (ICH and ischemic stroke)	CT head	Stroke neurologists reading CT	Single center
Beecy et al. (2017)	CNN	114	Stroke	CT head	Expert consensus interpretations	Single center
Dharmasaroja et al. (2013)	CNN	194	Stroke/intracranial hemorrhage	CT head	Thrombolysis after ischemic stroke	Single center
Fodeh et al. (2018)	SVM	1834	Atraumatic ICH	EHR review		Single center
Gottrup et al. (2005)	kNN, Custom-built algorithm	14	Acute ischemic stroke	MRI	Applicability of highly flexible instance-based methods	Single center
Ho et al. (2016)	SVM, RF, and GBRT models	105	Acute ischemic stroke	MRI	Classification models for the problem of unknown time-since-stroke	Single center
Knight-Greenfield et al. (2018)	CNN	114	Stroke	CT head	Expert consensus interpretations	Single center
Ramos et al. (2018)	SVM, RF, Logistic-linear regression, CNN	317	SAH	CT Head	Delayed cerebral ischemia in SAH detection	Single center
SÜt et al. (2012)	MLP neural networks	584	Stroke mortality	EMR data	Selected variables using univariate statistical analyses	N/A
Paula De Toledo et al. (2009)	Logistic-linear regression	441	SAH	CT Head	Algorithms used were C4.5, fast decision tree learner, partial decision trees, repeated incremental pruning to produce error reduction, nearest neighbor with generalization, and ripple down rule learner	Multicenter Register
Thorpe et al. (2018)	decision tree	66	Stroke	Transcranial Doppler	Velocity Curvature Index (VCI) vs Velocity Asymmetry Index (VAI)	Single center
Williamson et al. (2019)	BOOSTING algorithm, RF	483	Risk stratification in SAH	True poor outcomes		Single center
Xie et al. (2019)	Boosting algorithm	512	Predict Patient Outcome in Acute Ischemic Stroke	CT Head and clinical parameters	Feature selections were performed using a greedy algorithm	Single center
**Heart failure**						
Andjelkovic et al. (2014)	CNN	193	HF in congenital heart disease	Echocardiography		Single center
Blecker et al. (2018)	Logistic-linear regression	37,229	ADHF	Early ID of patients at risk of readmission for ADHF	4 algorithms tested	Single center
Gleeson et al. (2016)	Custom-built algorithm	534	HF	Echocardiography and ECG	Data mining was applied to discover novel ECG and echocardiographic markers of risk	Single center
Golas et al. (2018)	Logistic-linear regression, boosting algorithm, CNN	11,510	HF	EHR	Heat failure patients to predict 30 day readmissions	Several hospitals in the Partners Healthcare System
Mortazavi et al. (2016)	Random forests, boosting, combined algorithms or logistic regression	1653	HF	Surveys to hospital examinations		Tele-HF trial
Frizzell et al	Random forest and gradient-boosted algorithms	56,477	HF	EHR	Traditional statistical methods	GWTG-HF registry
Kasper Rossing et al. (2016)	SVM	33	HFpEF	Urinary proteomic analysis		Heart failure clinic (Single center)
Kiljanek et al. (2009)	RF	1587	HF	Clinical diagnosis	Development of congestive heart failure after NSTEMI	CRUSADE registry
Liu et al. (2016)	Boosting algorithm, Logistic-linear regression	526	HF	Medical data, blood test, and echocardiographic imaging	Predicting mortality in HF	Single center

*SVM* support vector machine, *RF* random forest, *CNN* convolutional neural network, *kNN* k-nearest neighbors, *PCA* principal component analysis, *GBRT* gradient boosted regression trees, *MLP* multilayer perceptron, *HER* electronic health record, *HF* heart failure, *HFpEF* heart failure with preserved ejection fraction, *ADHF* acute decompensated heart failure, *SAH* subarachnoid hemorrhage, *SDH* subdural hematoma, *ICH* intracerebral hemorrhage, *CAD* coronary artery disease, *ACS* acute coronary syndrome, *CCTA* coronary computed tomography angiography, *FFR* fractional flow reserve, *IVUS* intravascular ultrasound, *ICD* implantable cardioverter-defibrillator, *AF* atrial fibrillation, *ECG* electrocardiogram.

### ML algorithms and prediction of CAD

**Figure 2:**
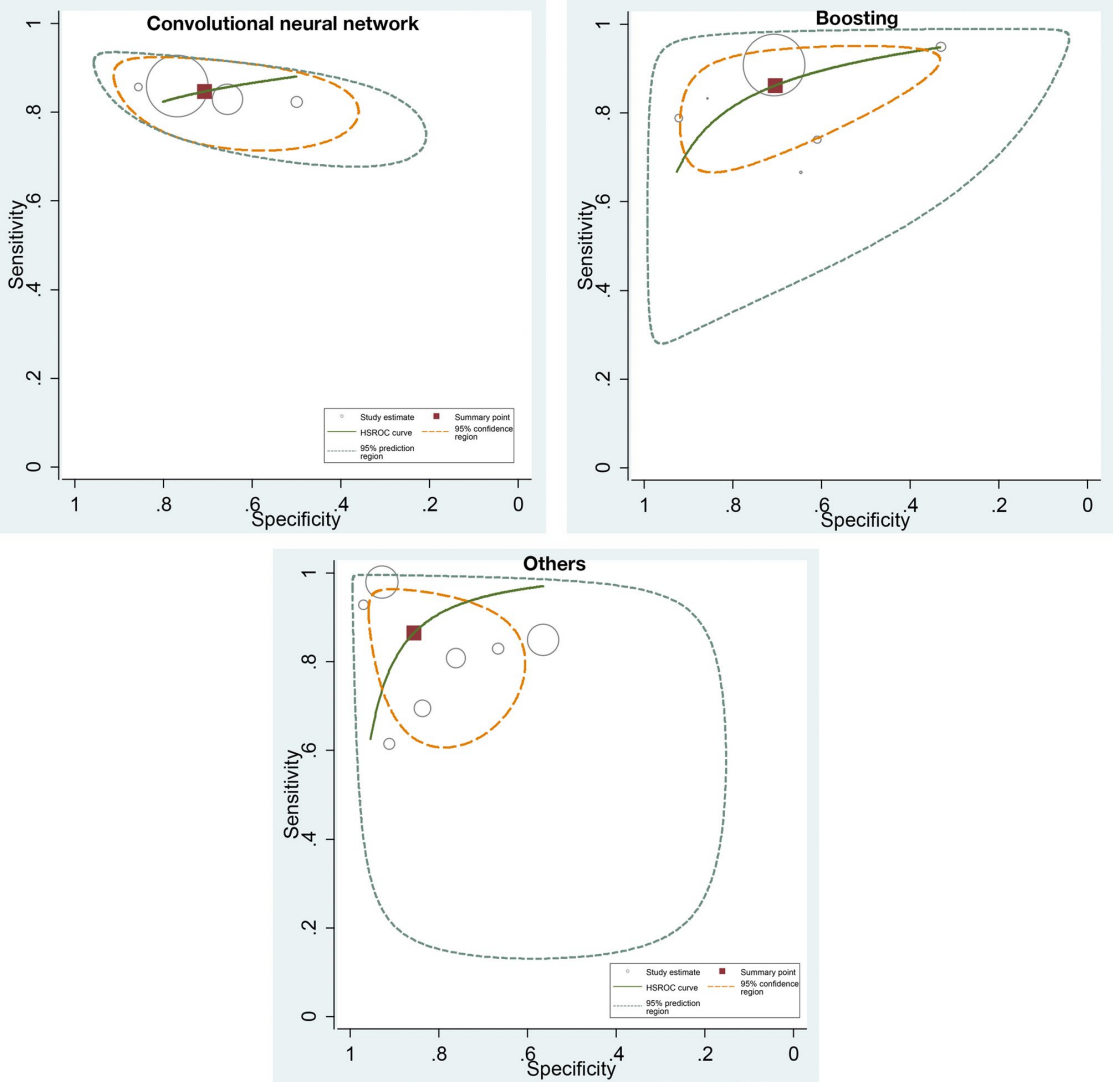
ROC curves comparing different machine learning models for CAD prediction. The prediction in CAD was associated with pooled AUC of 0.87 (95% CI 0.76–0.93) for CNN, pooled AUC of 0.88 (95% CI 0.84–0.91) for boosting algorithms, and pooled of AUC 0.93 (95% CI 0.85–0.97) for others (custom-built algorithms).

For the CAD, 45 cohorts reported a total of 116,227 individuals. 10 cohorts used CNN algorithms, 7 cohorts used SVM, 13 cohorts used boosting algorithm, 9 cohorts used custom-built algorithms, and 2 cohorts used RF. The prediction in CAD was associated with pooled AUC of 0.88 (95% CI 0.84–0.91), sensitivity of 0.86 (95% CI 0.77–0.92), and specificity of 0.70 (95% CI 0.51–0.84), for boosting algorithms and pooled of AUC 0.93 (95% CI 0.85–0.97), sensitivity of 0.87 (95% CI 0.74–0.94), and specificity of 0.86 (95% CI 0.73–0.93) for custom-built algorithms (Fig. 2).

### ML algorithms and prediction of stroke

**Figure 3:**
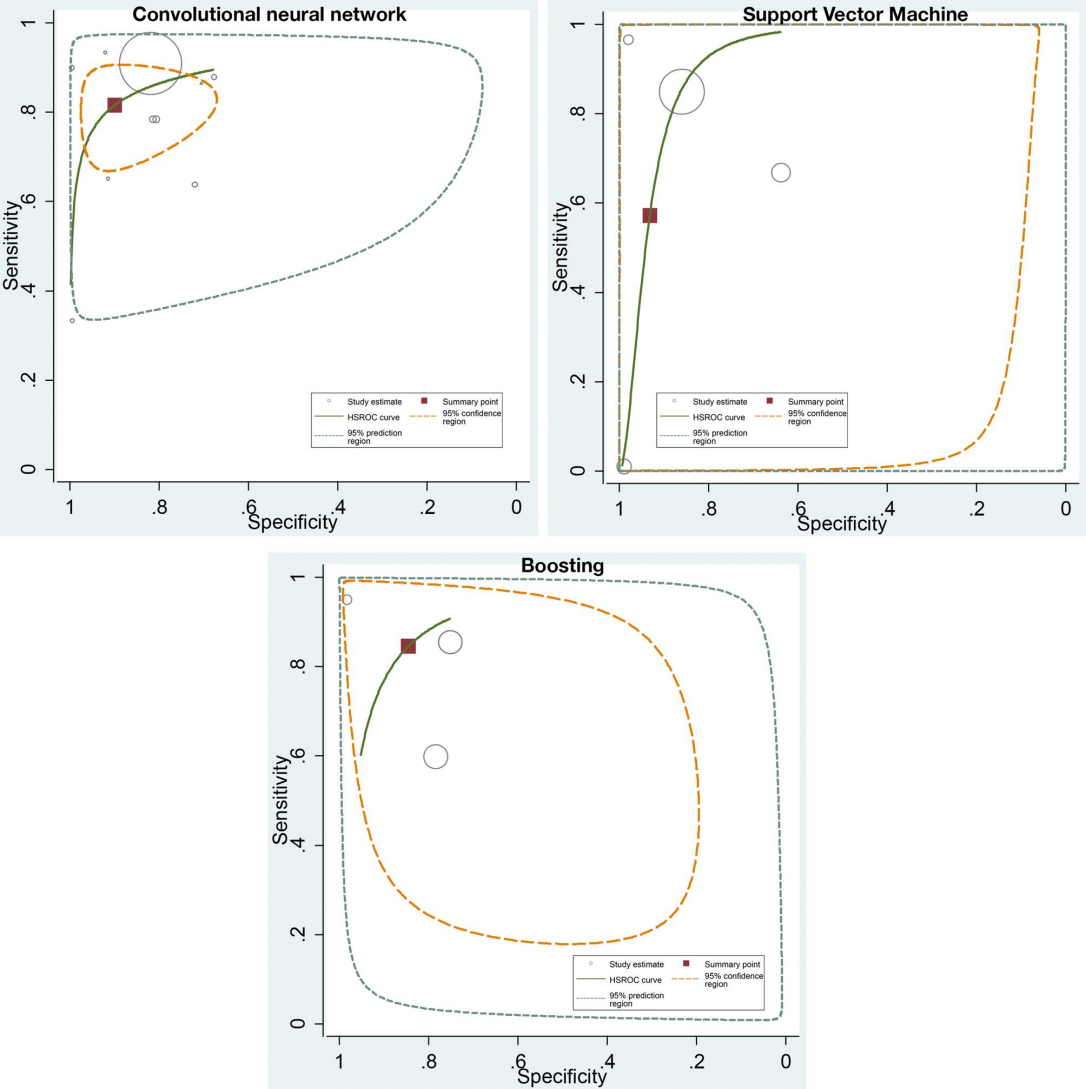
ROC curves comparing different machine learning models for stroke prediction. The prediction in stroke was associated with pooled AUC of 0.90 (95% CI 0.83–0.95) for CNN, pooled AUC of 0.92 (95% CI 0.81–0.97) for SVM algorithms, and pooled AUC of 0.91 (95% CI 0.81–0.96) for boosting algorithms.

For the stroke, 34 cohorts reported a total of 7,027 individuals. 14 cohorts used CNN algorithms, 4 cohorts used SVM, 5 cohorts used boosting algorithm, 2 cohorts used decision tree, 2 cohorts used custom-built algorithms, and 1 cohort used random forest (RF). For prediction of stroke, SVM algorithms had a pooled AUC of 0.92 (95% CI 0.81–0.97), sensitivity 0.57 (95% CI 0.26–0.96), and specificity 0.93 (95% CI 0.71–0.99); boosting algorithms had a pooled AUC of 0.91 (95% CI 0.81–0.96), sensitivity 0.85 (95% CI 0.66–0.94), and specificity 0.85 (95% CI 0.67–0.94); and CNN algorithms had a pooled AUC of 0.90 (95% CI 0.83–0.95), sensitivity of 0.80 (95% CI 0.70–0.87), and specificity of 0.91 (95% CI 0.77–0.97) (Fig. 3).

### ML algorithms and prediction of HF

For the HF, 12 cohorts reported a total of 51,612 individuals. 3 cohorts used CNN algorithms, 4 cohorts used logistic regression, 2 cohorts used boosting algorithm, 1 cohort used SVM, 1 cohort used in-house algorithm, and 1 cohort used RF. We could not perform analyses because we had too few studies (≤ 5) for each model.

### ML algorithms and prediction of cardiac arrhythmias

For the cardiac arrhythmias, 12 cohorts reported a total of 3,204,837 individuals. 2 cohorts used CNN algorithms, 2 cohorts used logistic regression, 3 cohorts used SVM, 1 cohort used k-NN algorithm, and 4 cohorts used RF. We could not perform analyses because we had too few studies (≤ 5) for each model.

## Discussion

To the best of our knowledge, this is the first and largest novel meta-analytic approach in ML research to date, which drew from an extensive number of studies that included over one million participants, reporting ML algorithms prediction in cardiovascular diseases. Risk assessment is crucial for the reduction of the worldwide burden of CVD. Traditional prediction models, such as the Framingham risk score^12^, the PCE model^13^, SCORE^14^, and QRISK^15^ have been derived based on multiple predictive factors. These prediction models have been implemented in guidelines; specifically, the 2010 American College of Cardiology/American Heart Association (ACC/AHA) guideline^16^ recommended the Framingham Risk Score, the United Kingdom National Institute for Health and Care Excellence (NICE) guidelines recommend the QRISK3 score^17^, and the 2016 European Society of Cardiology (ESC) guidelines recommended the SCORE model^18^. These traditional CVD risk scores have several limitations, including variations among validation cohorts, particularly in specific populations such as patients with rheumatoid arthritis^19,20^. Under some circumstances, the Framingham score overestimates CVD risk, potentially leading to overtreatment^20^. In general, these risk scores encompass a limited number of predictors and omit several important variables. Given the limitations of the most widely accepted risk models, more robust prediction tools are needed to more accurately predict CVD burden. Advances in computational power to process large amounts of data has accelerated interest in ML-based risk prediction, but clinicians typically have limited understanding of this methodology. Accordingly, we have taken a meta-analytic approach to clarify the insights that ML modeling can provide for CVD research.

Unfortunately, we do not know how or why the authors of the analyzed studies selected the chosen algorithms from the large array of options available. Researchers/authors may have selected potential models for their databases and performed several models (e.g., running parallel, hyperparameter tuning) while only reporting the best model, resulting in overfitting to their data. Therefore, we assume the AUC of each study is based upon the best possible algorithm available to the associated researchers. Most importantly, pooled analyses indicate that, in general, ML algorithms are accurate (AUC 0.8–0.9 s) in overall cardiovascular disease prediction. In subgroup analyses of each ML algorithms, ML algorithms are accurate (AUC 0.8–0.9 s) in CAD and stroke prediction. To date, only one other meta-analysis of the ML literature has been reported, and the underlying concept was similar to ours. The investigators compared the diagnostic performance of various deep learning models and clinicians based on medical imaging (2 studies pertained to cardiology)^21^. The investigators concluded that deep learning algorithms were promising but identified several methodological barriers to matching clinician-level accuracy^21^. Although our work suggests that boosting models and support vector machine (SVM) models are promising for predicting CAD and stroke risk, further study comparing human expert and ML models are needed.

First, the results showed that custom-built algorithms tend to perform better than boosting algorithm for CAD prediction in terms of AUC comparison. However, there is significant heterogeneity among custom-built algorithms that do not disclose their details. The boosting algorithm has been increasingly utilized in modern biomedicine^22,23^. In order to implement in clinical practice, the essential stages of designing a model and interpretation need to be uniform^24^. For implementation in clinical practice, custom-built algorithms must be transparent and replicated in multiple studies using the same set of independent variables.

Second, the result showed that boosting algorithms and SVM provides similar pooled AUC for stroke prediction. SVMs and boosting shared a common margin to address the clinical question. SVM seems to perform better than boosting algorithms in patients with stroke perhaps due to discrete, linear data or a proper non-linear kernel that fits the data better with improved generalization. SVM is an algorithm designed for maximizing a particular mathematical function with respect to a given collection of data. Compared to the other ML methods, SVM is more powerful at recognizing hidden patterns in complicated clinical datasets^2,25^. Both boosting and SVM algorithms have been widely used in biomedicine and prior studies showed mixed results^26–30^. SVM seems to outperform boosting in image recognition tasks^28^, while boosting seems to be superior in omic tasks^27^. However, in subgroup analysis, using research questions or types of protocols or images showed no difference in algorithm predictions.

Third, for heart failure and cardiac arrhythmias, we could not perform meta-analytic approaches due to the small number of studies for each model. However, based on our observation in our systematic review, SVM seems to outperform other predictive algorithms in detecting cardiac arrhythmias, especially in one large study^31^. Interestingly, in HF, the results are inconclusive. One small study showed promising results from SVM^32^. CNN seems to outperform others, but the results are suboptimal^33^. Although we assumed all reported algorithms have optimal variables, technical heterogeneity exists in ML algorithms (e.g., number of folds for cross-validation, bootstrapping techniques, how many run time [epochs], multiple parameters adjustments). In addition, optimal cut off for AUC remained unclear in clinical practice. For example, high or low sensitivity/specificity for each test depends on clinical judgement based on clinically correlated. In general, very high AUCs (0.95 or higher) are recommended, and it is known that AUC 0.50 is not able to distinguish between true and false. In some fields such as applied psychology^34^, with several influential variables, AUC values of 0.70 and higher would be considered strong effects. Moreover, standard practice for ML practitioners recommended reporting certain measures (e.g., AUC, c-statistics) without optimal sensitivity and specificity or model calibration, while interpretation in clinical practice is challenging. For example, the difference in BNP cut off for HF patients could result in a difference in volume management between diuresis and IV fluid in pneumonia with septic shock.

Compared to conventional risk scores, most ML models shared a common set of independent demographic variables (e.g., age, sex, smoking status) and include laboratory values. Although those variables are not well-validated individually in clinical studies, they may add predictive value in certain circumstances. Head-to-head studies comparing ML algorithms and conventional risk models are needed. If these studies demonstrate an advantage of ML-based prediction, the optimal algorithms could be implemented through electronic health records (EHR) to facilitate application in clinical practice. The EHR implementation is well poised for ML based prediction since the data are readily accessible, mitigating dependency on a large number of variables, such as discrete laboratory values. While it may be difficult for physicians in resource-constrained practice settings to access the input data necessary for ML algorithms, it is readily implemented in more highly developed clinical environments.

To this end, the selection of ML algorithm should base on the research question and the structure of the dataset (how large the population is, how many cases exist,  how balanced the dataset is,  how many available variables there are, whether the data is longitudinal or not, if the clinical outcome is binary or time to event, etc.) For example, CNN is particularly powerful in dealing with image data, while SVM can reduce the high dimensionality of the dataset if the kernel is correctly chosen. While when the sample size is not large enough, deep learning methods will likely overfit the data.  Most importantly, this study's intent is not to identify one algorithm that is superior to others.

## Limitations

Although the performance of ML-based algorithms seems satisfactory, it is far from optimal. Several methodological barriers can confound results and increase heterogeneity. First, technical parameters such as hyperparameter tuning in algorithms are usually not disclosed to the public, leading to high statistical heterogeneity. Indeed, heterogeneity measures the difference in effect size between studies. Therefore, in the present study, heterogeneity is inevitable as several factors can lead to this (e.g., fine-tuning models, hyperparameter selection, epochs). It is also a not good indicator to use as, in our HSROC model, we largely controlled the heterogeneity. Second, the data partition is also arbitrary because of no standard guidelines for utilization. In the present study, most included studies use 80/20 or 70/30 for training and validation sets. In addition, since the sample size for each type of CVD is small, the pooled results could potentially be biased. Third, feature selection methodologies, and techniques are arbitrary and heterogeneous. Fourth, due to the ambiguity of custom-built algorithms, we could not classify the type of those algorithms. Fifth, studies report different evaluation matrices (e.g., some did not report positive or negative cases, sensitivity/specificity, F-score, etc.). We did not report the confusion matrix for this meta-analytic approach as it required aggregation of raw numbers from studies without adjusting for difference between studies, which could result in bias. Instead, we presented pooled sensitivity and specificity using the HSROC model. Although ML algorithms are robust, several studies did not report complete evaluation metrics such as positive or negative cases, Beyes, bias accuracy, or analysis in the validation cohort since there are many ways to interpret the data  depending on the clinical context. Most importantly, some analyses did not correlate with the clinical context, which made it more difficult to interpret. The efficacy of meta-analysis is to increase the power of the study by using the same algorithms. In addition, clinical data are heterogeneous and usually imbalanced. Most ML research did not report balanced accuracy, which could mislead the readers. Sixth, we did not register the analysis in PROSPERO. Finally, some studies reported only the technical aspect without clinical aspects, likely due to a lack of clinician supervision.

## Conclusion

Although there are several limitations to overcome to be able to implement ML algorithms in clinical practice, overall ML algorithms showed promising results. SVM and boosting algorithms are widely used in cardiovascular medicine with good results. However, selecting the proper algorithms for the  appropriate research questions, comparison to human experts, validation cohorts, and reporting of  all possible evaluation matrices are needed for study interpretation in the correct clinical context. Most importantly, prospective studies comparing ML algorithms to conventional risk models are needed. Once validated in that way, ML algorithms could be integrated with electronic health record systems and applied in clinical practice, particularly in high resources areas.

## Supplementary information

Supplementary file1

## References

[1] Noble WS (2004). Support vector machine applications in computational biology. Kernel Methods Comput. Biol..

[2] Aruna S, Rajagopalan S (2011). A novel SVM based CSSFFS feature selection algorithm for detecting breast cancer. Int. J. Comput. Appl..

[3] Lakhani P, Sundaram B (2017). Deep learning at chest radiography: Automated classification of pulmonary tuberculosis by using convolutional neural networks. Radiology.

[4] Yasaka K, Akai H (2018). Deep learning with convolutional neural network for differentiation of liver masses at dynamic contrast-enhanced CT: A preliminary study. Radiology.

[5] Christ PF, Elshaer MEA, Ettlinger F (2016). Automatic Liver and Lesion Segmentation in CT Using Cascaded Fully Convolutional Neural Networks and 3D Conditional Random Fields. International Conference on Medical Image Computing and Computer-Assisted Intervention.

[6] Krittanawong C, Johnson KW, Rosenson RS (2019). Deep learning for cardiovascular medicine: A practical primer. Eur. Heart J..

[7] Krittanawong C, Zhang H, Wang Z, Aydar M, Kitai T (2017). Artificial intelligence in precision cardiovascular medicine. J. Am. Coll. Cardiol..

[8] Krittanawong C, Bomback AS, Baber U, Bangalore S, Messerli FH, Wilson Tang WH (2018). Future direction for using artificial intelligence to predict and manage hypertension. Curr. Hypertens. Rep..

[9] Covidence systematic review software. Melbourne AVHIAawcoAD.

[10] Rutter CM, Gatsonis CA (2001). A hierarchical regression approach to meta-analysis of diagnostic test accuracy evaluations. Stat. Med..

[11] Stroup DF, Berlin JA, Morton SC (2000). Meta-analysis of observational studies in epidemiology: A proposal for reporting. Meta-analysis Of Observational Studies in Epidemiology (MOOSE) group. JAMA.

[12] Wilson PW, D'Agostino RB, Levy D, Belanger AM, Silbershatz H, Kannel WB (1998). Prediction of coronary heart disease using risk factor categories. Circulation.

[13] Goff DC, Lloyd-Jones DM, Bennett G (2014). 2013 ACC/AHA guideline on the assessment of cardiovascular risk: A report of the American College of Cardiology/American Heart Association Task Force on Practice Guidelines. J. Am. Coll. Cardiol..

[14] Conroy RM, Pyörälä K, Fitzgerald AP (2003). Estimation of ten-year risk of fatal cardiovascular disease in Europe: The SCORE project. Eur. Heart J..

[15] Hippisley-Cox J, Coupland C, Vinogradova Y (2008). Predicting cardiovascular risk in England and Wales: Prospective derivation and validation of QRISK2. BMJ (Clinical research ed).

[16] Greenland P, Alpert JS, Beller GA (2010). 2010 ACCF/AHA guideline for assessment of cardiovascular risk in asymptomatic adults: A report of the American College of Cardiology Foundation/American Heart Association Task Force on Practice Guidelines. Circulation.

[17] Hippisley-Cox J, Coupland C, Brindle P (2017). Development and validation of QRISK3 risk prediction algorithms to estimate future risk of cardiovascular disease: Prospective cohort study. BMJ (Clinical research ed).

[18] Piepoli MF, Hoes AW, Agewall S (2016). 2016 European Guidelines on cardiovascular disease prevention in clinical practice: The Sixth Joint Task Force of the European Society of Cardiology and Other Societies on Cardiovascular Disease Prevention in Clinical Practice (constituted by representatives of 10 societies and by invited experts) Developed with the special contribution of the European Association for Cardiovascular Prevention & Rehabilitation (EACPR). Eur. Heart J..

[19] Kremers HM, Crowson CS, Therneau TM, Roger VL, Gabriel SE (2008). High ten-year risk of cardiovascular disease in newly diagnosed rheumatoid arthritis patients: A population-based cohort study. Arthritis Rheum..

[20] Damen JA, Pajouheshnia R, Heus P (2019). Performance of the Framingham risk models and pooled cohort equations for predicting 10-year risk of cardiovascular disease: A systematic review and meta-analysis. BMC Med..

[21] Liu X, Faes L, Kale AU (2019). A comparison of deep learning performance against health-care professionals in detecting diseases from medical imaging: A systematic review and meta-analysis. Lancet Digit. Health.

[22] Mayr A, Binder H, Gefeller O, Schmid M (2014). The evolution of boosting algorithms. From machine learning to statistical modelling. Methods Inf. Med..

[23] Buhlmann P, Gertheiss J, Hieke S (2014). Discussion of "the evolution of boosting algorithms" and "extending statistical boosting". Methods Inf. Med..

[24] Natekin A, Knoll A (2013). Gradient boosting machines, a tutorial. Front. Neurorobot..

[25] Noble WS (2006). What is a support vector machine?. Nat. Biotechnol..

[26] Zhang H, & Gu C. Support vector machines versus Boosting.

[27] Ogutu JO, Piepho HP, Schulz-Streeck T (2011). A comparison of random forests, boosting and support vector machines for genomic selection. BMC Proc..

[28] Sun T, Wang J, Li X (2013). Comparative evaluation of support vector machines for computer aided diagnosis of lung cancer in CT based on a multi-dimensional data set. Comput. Methods Programs Biomed..

[29] Huang M-W, Chen C-W, Lin W-C, Ke S-W, Tsai C-F (2017). SVM and SVM ensembles in breast cancer prediction. PLoS One.

[30] Caruana R, Karampatziakis N, & Yessenalina A. An empirical evaluation of supervised learning in high dimensions. In *Proceedings of the 25th International Conference on Machine Learning: ACM*, 2008, 96–103.

[31] Hill NR, Ayoubkhani D, Lumley M (2018). Machine learning to detect and diagnose atrial fibrillation and atrial flutter (AF/F) using routine clinical data. Value Health.

[32] Rossing K, Bosselmann HS, Gustafsson F (2016). Urinary proteomics pilot study for biomarker discovery and diagnosis in heart failure with reduced ejection fraction. PLoS One.

[33] Golas SB, Shibahara T, Agboola S (2018). A machine learning model to predict the risk of 30-day readmissions in patients with heart failure: A retrospective analysis of electronic medical records data. BMC Med. Inform. Decis. Mak..

[34] Rice ME, Harris GT (2005). Comparing effect sizes in follow-up studies: ROC Area, Cohen's d, and r. Law Hum Behav..

