# Supplementary material for: Machine learning prediction in cardiovascular diseases: a meta-analysis
Source: Sci Rep. 2020 Sep 29;10:16057. doi: 10.1038/s41598-020-72685-1 (PMC7525515; doi:10.1038/s41598-020-72685-1)
Supplement: Supplementary file 1 — Supplementary file1 [file 41598_2020_72685_MOESM1_ESM.docx]

**Title:** Machine Learning Prediction in Cardiovascular Diseases: A Meta-analysis

**Running Title:** Machine Learning Prediction in Cardiovascular Diseases

**Author list:** Chayakrit Krittanawong, MD^1,9^, Hafeez Ul Hassan Virk, MD^2^, Sripal Bangalore, MD, MHA^3^, Zhen Wang, PhD^4,5^, Kipp W. Johnson, PhD^6^, Rachel Pinotti, MLIS, AHIP^7^, HongJu Zhang, PhD^8^, Scott Kaplin, MD^9^, Bharat Narasimhan, MD^9^, Takeshi Kitai, MD, PhD^10^, Usman Baber, MD, MS^9^, Jonathan L Halperin, MD^9^, W.H. Wilson Tang, MD^10^

**Author Affiliations:**

^1^Section of Cardiology, Baylor College of Medicine, Houston, TX

^2^Department of Cardiovascular Diseases, Einstein Medical Center, Philadelphia, PA,

^3^Department of Cardiovascular Diseases, New York University School of Medicine

^4^Robert D. and Patricia E. Kern Center for the Science of Health Care Delivery,

^5^Division of Health Care Policy and Research, Department of Health Sciences Research,

^6^Institute for Next Generation Healthcare, Department of Genetics and Genomic Sciences, Icahn School of Medicine at Mount Sinai, New York, NY,

^7^Levy Library, Icahn School of Medicine at Mount Sinai, New York, NY,

^8^Division of Cardiovascular Diseases, Mayo Clinic, Rochester, MN,

^9^Department of Cardiovascular Diseases, Icahn School of Medicine at Mount Sinai, Mount Sinai Hospital, Mount Sinai Heart, New York, NY,

^10^Department of Cardiovascular Medicine, Heart and Vascular Institute, Cleveland Clinic, Cleveland, Ohio, USA

**Acknowledgements:** None

**Funding/Support:** There was no funding for this work.

**Conflict of Interest:** none declared.

**Word counts:** 3,174

Corresponding author:

Chayakrit Krittanawong, MD

Baylor College of Medicine

Section of Cardiology

1 Baylor Plaza, Houston, TX 77030

Tel: 713-798-4951

Email: [Chayakrit.Krittanawong@bcm.edu](mailto:Chayakrit.Krittanawong@bcm.edu)

**Keywords:** Machine learning; Cardiovascular disease; Systematic review; Meta-analysis

**Supplementary Material**

**Methods 1:** Search Strategies

**Methods 2:** Study Selection Strategies

**Table 1:** quality assessment of included studies in the meta-analysis

**Methods 1:** Search Strategies

1. exp cardiovascular disease/

2. (cardio* or cardia* or heart* or coronary* or angina* or ventric* or myocard* or pericard* or isch?em* or arrhythmi* or atrial fibrillat* or tachycardi* or endocardi*).tw.

3. exp cerebrovascular disease/

4. (stroke* or cerebrovasc* or cerebral vascular or apoplexy).tw.

5. ((brain* or cerebral or lacunar) adj2 (infarct* or accident*)).tw.

6. exp hypertension/

7. (hypertensi* or peripheral arter* disease*).tw.

8. ((high or increased or elevated) adj2 blood pressure).tw.

9. exp hyperlipidemia/

10. (hyperlipid* or hyperlip?emia* or hypercholesterol* or hypercholester?emia* or hyperlipoprotein?emia* or hypertriglycerid?emia*).

11. arteriosclerosis/ or arteriolosclerosis/ or atherosclerosis/

12. (arterio?sclerosis or atherosclerosis).tw.

13. 1 or 2 or 3 or 4 or 5 or 6 or 7 or 8 or 9 or 10 or 11 or 12

14. artificial neural network/ or machine learning/ or expert system/ or artificial intelligence/

15. ((machine or deep) adj2 learning).tw.

16. (neural networks or artificial intelligence or cognitive computing).tw.

17. (computer adj2 aided adj5 (diagnos* or decision*)).tw.

18. 14 or 15 or 16 or 17

19. 13 and 18

**Methods 2:** Study Selection Strategies

Machine learning studies were considered eligible if they (1) provided information related to machine learning algorithms; (2) reported one of performance measures (AUC, sensitivity, specificity, positive case (the number of patients who used the AI and were positively diagnosed with disease), negative (the number of patients using the AI who were negative according to the AI), true positive, false positive, true negative, or false negative; (3) provided information on cardiovascular outcomes including coronary heart disease, stroke, heart failure or cardiac arrhythmias; and (4) were published in the English language.

**Table 1:** Risk of bias assessment of included studies in the meta-analysis

| **First author** | **Algorithms** | **Resource** | **Data** | **Ground truth** | **Outcome** | **Interpretation** | **Summary** |
| --- | --- | --- | --- | --- | --- | --- | --- |
| **Cardiac arrhythmias** | | | | | | | |
| Alickovic et al.[^1^](#_ENREF_1) (2016) | 1 | 2 | 1 | 0 | 1 | 1 | 6 |
| Au-Yeung et al.[^2^](#_ENREF_2) (2018) | 3 | 2 | 2 | 0 | 2 | 2 | 11 |
| Hill et al.[^3^](#_ENREF_3) (2018) | 3 | 2 | 0 | 0 | 1 | 1 | 7 |
| Kotu et al.[^4^](#_ENREF_4) (2015) | 3 | 1 | 1 | 0 | 1 | 1 | 7 |
| Ming-Zher Poh et al.[^5^](#_ENREF_5) (2018) | 3 | 2 | 1 | 0 | 3 | 2 | 11 |
| Xiaoyan Xu et al.[^6^](#_ENREF_6) (2018) | 1 | 1 | 1 | 0 | 2 | 2 | 7 |
| **Coronary artery disease** | | | | | | | |
| Araki et al.[^7^](#_ENREF_7) (2016) | 1 | 1 | 1 | 0 | 1 | 1 | 5 |
| Araki et al.[^8^](#_ENREF_8) (2016) | 1 | 1 | 1 | 0 | 1 | 1 | 5 |
| Arsanjani et al.[^9^](#_ENREF_9) (2013) | 1 | 1 | 2 | 1 | 1 | 1 | 7 |
| Baumann et al.[^10^](#_ENREF_10) (2017) | 1 | 1 | 0 | 0 | 1 | 1 | 4 |
| Coenen et al.[^11^](#_ENREF_11) (2018) | 1 | 2 | 2 | 0 | 2 | 2 | 9 |
| Dey et al.[^12^](#_ENREF_12) (2015) | 2 | 1 | 2 | 0 | 2 | 2 | 9 |
| Eisenberg et al.[^13^](#_ENREF_13) (2018) | 2 | 2 | 0 | 0 | 1 | 1 | 6 |
| Freiman et al.[^14^](#_ENREF_14) (2017) | 1 | 1 | 1 | 0 | 1 | 1 | 5 |
| Guner et al.[^15^](#_ENREF_15) (2010) | 1 | 1 | 2 | 0 | 3 | 2 | 9 |
| Hae et al.[^16^](#_ENREF_16) (2018) | 1 | 1 | 2 | 0 | 2 | 2 | 8 |
| Han et al.[^17^](#_ENREF_17) (2017) | 1 | 1 | 2 | 0 | 1 | 2 | 7 |
| Hu (Xiuhua) et al.[^18^](#_ENREF_18) (2018) | 1 | 2 | 0 | 0 | 1 | 1 | 5 |
| Hu et al.[^19^](#_ENREF_19) (2018) | 1 | 2 | 0 | 0 | 1 | 1 | 5 |
| Wei et al.[^20^](#_ENREF_20) (2014) | 1 | 2 | 1 | 0 | 1 | 1 | 6 |
| Kranthi et al.[^21^](#_ENREF_21) (2017) | 1 | 1 | 0 | 0 | 1 | 1 | 4 |
| Madan et al.[^22^](#_ENREF_22) (2013) | 1 | 1 | 0 | 0 | 1 | 1 | 4 |
| Zellweger et al.[^23^](#_ENREF_23) (2018) | 1 | 2 | 2 | 1 | 2 | 2 | 10 |
| Moshrik Abd alamir et al.[^24^](#_ENREF_24) (2018) | 1 | 1 | 2 | 0 | 2 | 2 | 8 |
| Nakajima et al.[^25^](#_ENREF_25) (2017) | 1 | 2 | 2 | 1 | 2 | 2 | 10 |
| Song et al.[^26^](#_ENREF_26) (2014) | 1 | 1 | 2 | 0 | 1 | 1 | 6 |
| VanHouten et al.[^27^](#_ENREF_27) (2014) | 3 | 1 | 2 | 1 | 2 | 2 | 11 |
| Xiao et al.[^28^](#_ENREF_28) (2018) | 1 | 1 | 1 | 0 | 1 | 2 | 6 |
| Yoneyama et al.[^29^](#_ENREF_29) (2017) | 1 | 1 | 2 | 0 | 2 | 2 | 8 |
| **Stroke** | | | | | | | |
| Abouzari et al.[^30^](#_ENREF_30) (2009) | 1 | 1 | 1 | 0 | 1 | 2 | 6 |
| Alexander Roederer et al.[^31^](#_ENREF_31) (2014) | 1 | 1 | 2 | 0 | 1 | 2 | 7 |
| Arslan et al.[^32^](#_ENREF_32) (2016) | 3 | 1 | 1 | 0 | 2 | 2 | 9 |
| Atanassova et al.[^33^](#_ENREF_33) (2008) | 1 | 1 | 2 | 0 | 1 | 1 | 6 |
| Barriera et al.[^34^](#_ENREF_34) (2018) | 1 | 1 | 0 | 0 | 1 | 1 | 4 |
| Beecy et al.[^35^](#_ENREF_35) (2017) | 1 | 1 | 0 | 1 | 1 | 2 | 6 |
| Dharmasaroja et al.[^36^](#_ENREF_36) (2013) | 3 | 1 | 2 | 0 | 1 | 1 | 8 |
| Fodeh et al.[^37^](#_ENREF_37) (2018) | 1 | 1 | 0 | 0 | 1 | 1 | 4 |
| Gottrup et al.[^38^](#_ENREF_38) (2005) | 3 | 1 | 1 | 0 | 1 | 1 | 7 |
| Ho et al.[^39^](#_ENREF_39) (2016) | 1 | 1 | 2 | 0 | 1 | 1 | 6 |
| Knight-Greenfield et al.[^40^](#_ENREF_40) (2018) | 1 | 1 | 0 | 0 | 1 | 1 | 4 |
| Ramos et al.[^41^](#_ENREF_41) (2018) | 3 | 1 | 1 | 0 | 2 | 2 | 9 |
| SÜt et al.[^42^](#_ENREF_42) (2012) | 1 | 1 | 2 | 0 | 1 | 2 | 7 |
| Paula De Toledo et al.[^43^](#_ENREF_43) (2009) | 1 | 2 | 2 | 0 | 3 | 1 | 9 |
| Thorpe et al.[^44^](#_ENREF_44) (2018) | 1 | 1 | 1 | 0 | 1 | 2 | 6 |
| Williamson et al. (2019) | 1 | 1 | 0 | 0 | 1 | 1 | 4 |
| Xie et al.[^45^](#_ENREF_45) (2019) | 2 | 1 | 1 | 0 | 1 | 1 | 6 |
| **Heart failure** | | | | | | | |
| Andjelkovic et al.[^46^](#_ENREF_46) (2014) | 1 | 1 | 0 | 0 | 1 | 1 | 4 |
| Blecker et al.[^47^](#_ENREF_47) (2018) | 1 | 1 | 2 | 0 | 2 | 1 | 7 |
| Gleeson et al.[^48^](#_ENREF_48) (2016) | 1 | 1 | 0 | 0 | 1 | 1 | 4 |
| Golas et al.[^49^](#_ENREF_49) (2018) | 3 | 2 | 2 | 0 | 2 | 2 | 11 |
| Kasper Rossing et al.[^50^](#_ENREF_50) (2016) | 1 | 1 | 2 | 0 | 1 | 2 | 7 |
| Kiljanek et al.[^51^](#_ENREF_51) (2009) | 1 | 2 | 0 | 0 | 1 | 1 | 5 |
| Liu et al.[^52^](#_ENREF_52) (2016) | 1 | 1 | 0 | 0 | 1 | 1 | 4 |

**REFERENCES**

1. Alickovic E, Subasi A. Medical Decision Support System for Diagnosis of Heart Arrhythmia using DWT and Random Forests Classifier. Apr 2016;40(4):108.

2. Au-Yeung WM, Reinhall PG, Bardy GH, Brunton SL. Development and validation of warning system of ventricular tachyarrhythmia in patients with heart failure with heart rate variability data. *PloS one.* 2018;13(11):e0207215.

3. Hill NR, Ayoubkhani D, Lumley M, et al. Machine Learning to Detect and Diagnose Atrial Fibrillation and Atrial Flutter (AF/F) Using Routine Clinical Data. *Value in Health.* 2018;21:S213.

4. Kotu LP, Engan K, Borhani R, et al. Cardiac magnetic resonance image-based classification of the risk of arrhythmias in post-myocardial infarction patients. *Artificial intelligence in medicine.* Jul 2015;64(3):205-215.

5. Poh M-Z, Poh YC, Chan P-H, et al. Diagnostic assessment of a deep learning system for detecting atrial fibrillation in pulse waveforms. *Heart.* 2018;104(23):1921.

6. Xu X, Wei S, Ma C, Luo K, Zhang L, Liu C. Atrial fibrillation beat identification using the combination of modified frequency slice wavelet transform and convolutional neural networks. *Journal of healthcare engineering.* 2018;2018.

7. Araki T, Ikeda N, Shukla D, et al. A new method for IVUS-based coronary artery disease risk stratification: A link between coronary & carotid ultrasound plaque burdens. *Computer methods and programs in biomedicine.* Feb 2016;124:161-179.

8. Araki T, Ikeda N, Shukla D, et al. PCA-based polling strategy in machine learning framework for coronary artery disease risk assessment in intravascular ultrasound: A link between carotid and coronary grayscale plaque morphology. *Computer methods and programs in biomedicine.* May 2016;128:137-158.

9. Arsanjani R, Xu Y, Dey D, et al. Improved accuracy of myocardial perfusion SPECT for detection of coronary artery disease by machine learning in a large population. *Journal of nuclear cardiology : official publication of the American Society of Nuclear Cardiology.* Aug 2013;20(4):553-562.

10. Baumann S, Renker M, Tesche C, et al. Abstract 16511: Gender Differences in the Diagnostic Performance of a Machine Learning Based CT Angiography Derived FFR Algorithm Insights From the MACHINE Registry. *Circulation.* 2017/11/14 2017;136(suppl_1):A16511-A16511.

11. Coenen A, Kim YH, Kruk M, et al. Diagnostic Accuracy of a Machine-Learning Approach to Coronary Computed Tomographic Angiography-Based Fractional Flow Reserve: Result From the MACHINE Consortium. *Circulation. Cardiovascular imaging.* Jun 2018;11(6):e007217.

12. Dey D, Diaz Zamudio M, Schuhbaeck A, et al. Relationship Between Quantitative Adverse Plaque Features From Coronary Computed Tomography Angiography and Downstream Impaired Myocardial Flow Reserve by 13N-Ammonia Positron Emission Tomography: A Pilot Study. *Circulation. Cardiovascular imaging.* Oct 2015;8(10):e003255.

13. Eisenberg E, Betancur J, Hu L-H, et al. The Diagnostic Accuracy of Machine Learning From Stress Only fast-MPS. *Journal of Nuclear Medicine.* May 1, 2018 2018;59(supplement 1):508.

14. Freiman M, Nickisch H, Prevrhal S, et al. Improving CCTA-based lesions' hemodynamic significance assessment by accounting for partial volume modeling in automatic coronary lumen segmentation. *Medical physics.* Mar 2017;44(3):1040-1049.

15. Guner LA, Karabacak NI, Akdemir OU, et al. An open-source framework of neural networks for diagnosis of coronary artery disease from myocardial perfusion SPECT. *Journal of nuclear cardiology : official publication of the American Society of Nuclear Cardiology.* Jun 2010;17(3):405-413.

16. Hae H, Kang SJ. Machine learning assessment of myocardial ischemia using angiography: Development and retrospective validation. Nov 2018;15(11):e1002693.

17. Han D, Lee JH, Rizvi A, et al. Incremental role of resting myocardial computed tomography perfusion for predicting physiologically significant coronary artery disease: A machine learning approach. *Journal of nuclear cardiology : official publication of the American Society of Nuclear Cardiology.* Feb 2018;25(1):223-233.

18. Hu X, Yang M, Han L, Du Y. Diagnostic performance of machine-learning-based computed fractional flow reserve (FFR) derived from coronary computed tomography angiography for the assessment of myocardial ischemia verified by invasive FFR. *The international journal of cardiovascular imaging.* Dec 2018;34(12):1987-1996.

19. Hu LH, Betancur J, Sharir T, et al. Machine learning predicts per-vessel early coronary revascularization after fast myocardial perfusion SPECT: results from multicentre REFINE SPECT registry. *European heart journal cardiovascular Imaging.* Jul 16 2019.

20. Wei J, Zhou C, Chan HP, et al. Computerized detection of noncalcified plaques in coronary CT angiography: evaluation of topological soft gradient prescreening method and luminal analysis. *Medical physics.* Aug 2014;41(8):081901.

21. Kolli Kranthi K, Han D, Gransar H, et al. Abstract 18581: Machine Learning Algorithms and Coronary Calcium Score for Risk Stratification in Asymptomatic Healthy Population. *Circulation.* 2017/11/14 2017;136(suppl_1):A18581-A18581.

22. Madan G SM, Sharma A, Kakkar VV, Vangala RK. Urinary Biomarker discovery for coronary artery disease risk prediction using global proteomic analysis. International Society on Thrombosis and Haemostasis 11 (Suppl. 2) (2013) 290–1019.

23. Zellweger MJ, Tsirkin A, Vasilchenko V, et al. A new non-invasive diagnostic tool in coronary artery disease: artificial intelligence as an essential element of predictive, preventive, and personalized medicine. *The EPMA journal.* Sep 2018;9(3):235-247.

24. Abd Alamir M, Noack P, Jang KH, Moore JA, Goldberg R, Poon M. Computer-aided analysis of 64- and 320-slice coronary computed tomography angiography: a comparison with expert human interpretation. *The international journal of cardiovascular imaging.* Sep 2018;34(9):1473-1483.

25. Nakajima K, Kudo T, Nakata T, et al. Diagnostic accuracy of an artificial neural network compared with statistical quantitation of myocardial perfusion images: a Japanese multicenter study. *European journal of nuclear medicine and molecular imaging.* Dec 2017;44(13):2280-2289.

26. Song T, Qu XF, Zhang YT, et al. Usefulness of the heart-rate variability complex for predicting cardiac mortality after acute myocardial infarction. *BMC cardiovascular disorders.* May 1 2014;14:59.

27. VanHouten JP, Starmer JM, Lorenzi NM, Maron DJ, Lasko TA. Machine learning for risk prediction of acute coronary syndrome. Paper presented at: AMIA Annual Symposium Proceedings2014.

28. Xiao R, Xu Y, Pelter MM, Mortara DW, Hu X. A Deep Learning Approach to Examine Ischemic ST Changes in Ambulatory ECG Recordings. *AMIA Joint Summits on Translational Science proceedings. AMIA Joint Summits on Translational Science.* 2018;2017:256-262.

29. Yoneyama H, Nakajima K, Taki J, et al. Ability of artificial intelligence to diagnose coronary artery stenosis using hybrid images of coronary computed tomography angiography and myocardial perfusion SPECT. *European Journal of Hybrid Imaging.* 2019;3(1):4.

30. Abouzari M, Rashidi A, Zandi-Toghani M, Behzadi M, Asadollahi M. Chronic subdural hematoma outcome prediction using logistic regression and an artificial neural network. *Neurosurgical review.* Oct 2009;32(4):479-484.

31. Roederer A, Holmes JH, Smith MJ, Lee I, Park S. Prediction of significant vasospasm in aneurysmal subarachnoid hemorrhage using automated data. *Neurocritical care.* Dec 2014;21(3):444-450.

32. Arslan AK, Colak C, Sarihan ME. Different medical data mining approaches based prediction of ischemic stroke. *Computer methods and programs in biomedicine.* Jul 2016;130:87-92.

33. Atanassova PA, Chalakova NT, Dimitrov BD. Diastolic blood pressure cut-off predicts major cerebrovascular events after minor ischaemic stroke: a post-hoc modelling study. *Central European Journal of Medicine.* 2008/10/22 2008;3(4):430.

34. Barreira C, Bouslama M, Ratcliff J, et al. E-078 Advance study: automated detection and volumetric assessment of intracerebral hemorrhage. *Journal of NeuroInterventional Surgery.* 2018;10(Suppl 2):A88-A88.

35. Beecy AN, Chang Q, Anchouche K, et al. A Novel Deep Learning Approach for Automated Diagnosis of Acute Ischemic Infarction on Computed Tomography. *JACC. Cardiovascular imaging.* Nov 2018;11(11):1723-1725.

36. Dharmasaroja P, Dharmasaroja PA. Prediction of intracerebral hemorrhage following thrombolytic therapy for acute ischemic stroke using multiple artificial neural networks. *Neurological research.* Mar 2012;34(2):120-128.

37. Fodeh S FE, Yip R, Littauer R, Sather J, Sheth KN, Matouk C, Parwani V, Ulrich A, Pham L, Venkatesh AK. Identification of patients with atraumatic intracranial hemorrhage: Novel Applications for Machine Learning. Society of Academic Emergency Medicine 2018 Annual Meeting, May 15-18, Indianapolis, In.

38. Gottrup C, Thomsen K, Locht P, et al. Applying instance-based techniques to prediction of final outcome in acute stroke. *Artificial intelligence in medicine.* Mar 2005;33(3):223-236.

39. Ho KC, Speier W, El-Saden S, Arnold CW. Classifying Acute Ischemic Stroke Onset Time using Deep Imaging Features. *AMIA ... Annual Symposium proceedings. AMIA Symposium.* 2017;2017:892-901.

40. Knight-Greenfield A, Beecy A, Chang Q, et al. Abstract TP58: A Novel Deep Learning Approach for Automated Diagnosis of Cerebral Infarction on Computed Tomography. *Stroke.*49(Suppl_1):ATP58-ATP58.

41. Ramos LA, van der Steen WE, Sales Barros R, et al. Machine learning improves prediction of delayed cerebral ischemia in patients with subarachnoid hemorrhage. *Journal of NeuroInterventional Surgery.* 2019;11(5):497.

42. Süt N, Çelik Y. Prediction of mortality in stroke patients using multilayer perceptron neural networks. *Turkish Journal of Medical Sciences.* 2012;42(5):886-893.

43. de Toledo P, Rios PM, Ledezma A, Sanchis A, Alen JF, Lagares A. Predicting the outcome of patients with subarachnoid hemorrhage using machine learning techniques. *IEEE transactions on information technology in biomedicine : a publication of the IEEE Engineering in Medicine and Biology Society.* Sep 2009;13(5):794-801.

44. Thorpe SG, Thibeault CM, Canac N, Wilk SJ, Devlin T, Hamilton RB. Decision Criteria for Large Vessel Occlusion Using Transcranial Doppler Waveform Morphology. *Frontiers in neurology.* 2018;9:847.

45. Xie Y, Jiang B, Gong E, et al. JOURNAL CLUB: Use of Gradient Boosting Machine Learning to Predict Patient Outcome in Acute Ischemic Stroke on the Basis of Imaging, Demographic, and Clinical Information. *AJR. American journal of roentgenology.* Jan 2019;212(1):44-51.

46. Andjelkovic K K-OI, Nedeljkovic D, Andjelkovic I. Role of echocardiography in prediction of heart failure in adults with congenital heart disease. Eur Heart J Cardiovasc Imaging. European Heart Journal - Cardiovascular Imaging. 2014;15(suppl_2):ii168-ii195.

47. Blecker S, Sontag D, Horwitz LI, et al. Early Identification of Patients With Acute Decompensated Heart Failure. *Journal of cardiac failure.* Jun 2018;24(6):357-362.

48. Gleeson S, Liao Y-W, Dugo C, et al. Machine Learning Applied to Advanced ECG and Echocardiographic Metadata, in Patients with Left Ventricular Dysfunction. *Heart, Lung and Circulation.* 2016;25:S10-S11.

49. Golas SB, Shibahara T, Agboola S, et al. A machine learning model to predict the risk of 30-day readmissions in patients with heart failure: a retrospective analysis of electronic medical records data. *BMC medical informatics and decision making.* Jun 22 2018;18(1):44.

50. Rossing K, Bosselmann HS, Gustafsson F, et al. Urinary Proteomics Pilot Study for Biomarker Discovery and Diagnosis in Heart Failure with Reduced Ejection Fraction. *PloS one.* 2016;11(6):e0157167.

51. Kiljanek LR, Cheriyath P. Prediction of Congestive Heart Failure as Adverse Outcome of Non-ST-Elevation Myocardial Infarction. *Journal of cardiac failure.* 2009;15(6):S93-S94.

52. Liu Y, Scirica BM, Stultz CM, Guttag JV. Beatquency domain and machine learning improve prediction of cardiovascular death after acute coronary syndrome. *Scientific reports.* Oct 6 2016;6:34540.
